# Prognostic Significance of MYH9 Expression in Resected Non-Small Cell Lung Cancer

**DOI:** 10.1371/journal.pone.0121460

**Published:** 2015-03-31

**Authors:** Ken Katono, Yuichi Sato, Shi-Xu Jiang, Makoto Kobayashi, Ryo Nagashio, Shinichiro Ryuge, Eriko Fukuda, Naoki Goshima, Yukitoshi Satoh, Makoto Saegusa, Noriyuki Masuda

**Affiliations:** 1 Department of Respiratory Medicine, School of Medicine, Kitasato University, Kanagawa, Japan; 2 Department of Molecular Diagnostics, School of Allied Health Sciences, Kitasato University, Kanagawa, Japan; 3 Department of Pathology, School of Medicine, Kitasato University, Kanagawa, Japan; 4 Division of Quantitative Proteomics Team, Molecular Profiling Research Center for Drug Discovery, National Institute of Advanced Industrial Science and Technology (AIST), Tokyo, Japan; 5 Department of Thoracic and Cardiovascular Surgery, School of Medicine, Kitasato University, Kanagawa, Japan; University General Hospital of Heraklion and Laboratory of Tumor Cell Biology, School of Medicine, University of Crete, GREECE

## Abstract

**Introduction:**

Myosin-9 (MYH9) belongs to the myosin superfamily of actin-binding motor protein. Recently, MYH9 has been thought to be associated with cancer cell migration, invasion, and metastasis. The aims of this study were to immunohistochemically examine MYH9 expression in surgically resected non-small cell lung cancer (NSCLC), and evaluate its correlations with clinicopathological parameters and the prognosis of patients.

**Methods:**

MYH9 expression was immunohistochemically studied in 266 consecutive resected NSCLCs, and its associations with clinicopathological parameters were evaluated. Kaplan-Meier survival analysis and Cox proportional hazards models were used to estimate the effect of MYH9 expression on survival.

**Results:**

MYH9 expression was detected in 102 of 266 (38.3%) NSCLCs. MYH9 expression was significantly correlated with the adenocarcinoma histology (*P* = 0.014), poorer differentiation ((*P* = 0.033), intratumoral vascular invasion and lymphatic invasion ((*P* = 0.013 and P = 0.045 respectively), and a poorer prognosis ((*P* = 0.032). In addition, multivariable analysis revealed that MYH9 expression independently predicted a poorer survival (HR, 2.15; 95%CI, 1.17-3.92; (*P* = 0.01).

**Conclusion:**

The present study revealed that MYH9 is expressed in a subset of NSCLC with a more malignant nature, and its expression is an indicator of a poorer survival probability.

## Introduction

Primary lung cancer is the leading cause of cancer-related mortality worldwide, with non-small cell lung cancer (NSCLC) accounting for nearly 80% of deaths [1]. The prognosis of patients with NSCLC is principally correlated with tumor metastasis. The process of tumor metastasis consists of complex steps: that involving tumor cell migration followed by detachment from the primary tumor, invasion into surrounding tissues, intravasation to blood or lymphatic vessels, dissemination in the hemolymphatic system, and extravasation at secondary sites [2]. Understanding proteins related to tumor migration, invasion, and metastasis may be useful as new prognostic markers or therapeutic targets in NSCLC. A close correlation between metastasis and chemoresistance was frequently observed in human cancer patients, some molecules which are related to metastasis and chemoresistance were reported [3,4,5]. Therefore, we focused proteins from cisplatin-resistant sub-lines to detect a novel marker which indicates aggressive clinical behavior in NSCLC.

In the present study, we focused on Myosin-9 (MYH9), which is increased in cisplatin-resistant sub-lines. The myosin superfamily is represented by fifteen classes. Myosin-II, the conventional two-headed myosin that forms bipolar filaments, is directly involved in regulating cytokinesis, cell motility, and cell morphology in nonmuscle cells. Vertebrates express at least two non-muscle myosin II heavy chain isoforms, referred to as myosin-IIA and myosin-IIB. The former consists of two non-muscle myosin IIA heavy chains (NMMHC-IIA), referred to as Myosin-9 (MYH9), two regulatory light chains (RLC), and two essential light chains. The activity of myosin-IIA is mainly regulated by the phosphorylation of RLC and MYH9 [6]. Because MYH9 contributes to cell polarity, adhesion, division, and migration [7,8], several studies have indicated that MYH9 plays a key role in cancer cell migration, invasion, and metastasis [9,10]. In human MCF-7 breast cancer cells, MCF-7/6 cells which have an invasive ability show a higher expression of MYH9 than MCF-A/Z cells, which are noninvasive. The invasiveness of MCF-7/6 is decreased either by knockdown of MYH9 or treatment with blebbistatin, which inhibits the function of MYH9, indicating the involvement of MYH9 in the migration and invasion of cancer cells. The overexpression of MYH9 is related to a poor prognosis in esophageal [11], bladder [12], and gastric cancer [13]. Maeda et al. reported that stage I patients with lung adenocarcinoma lacking the expression of either MYH9 or vimentin have a favorable outcome without postoperative adjuvant chemotherapy [14]. However, the relationships between MYH9 expression and clinicopathological features, and patients’ prognoses need to be further studied in a large number of NSCLC cases at various disease stages as well as in lung squamous cell carcinoma. The present study examined MYH9 expression in resected NSCLCs including squamous cell carcinomas of pathological stage I-III, and analyzed the correlation with clinicopathological parameters of patients and its prognostic significance.

## Materials and Methods

### Ethics statements

The study was approved by the Ethics Committee of the Kitasato University School of Medicine (B13-53) and followed the Declaration of Helsinki protocol. All patients were approached based on approved ethical guidelines, agreed to participate in this study, and could refuse entry and discontinue participation at any time. All participants proved written consent. Animals in this study were handled according to the Guidelines for Animal Experiments of Kitasato University School of Allied Health Sciences, and all animal experimental protocol was approved by the Committee on the ethics of Animal Experiments of the Kitasato University School of Allied Health Sciences (14–16).

### Cell lines

The LCN1, derived from large cell neuroendocrine carcinoma, was established in our laboratory [15]. The LC-2/ad and A549, both derived from a lung adenocarcinoma, were purchased from the RIKEN BioResource Center (Ibaraki, Japan) and Japanese Cancer Research Resources Bank (Tokyo, Japan), respectively. These cells were grown in RPMI-1640 medium (SIGMA, Steinheim, Germany) supplemented with 10% fetal bovine serum (FBS; Biowest, Miami, FL, USA), 100 units/ml of penicillin, and 100 μg/ml of streptomycin (GIBCO, Auckland, New Zealand). After harvesting and washing twice with phosphate-buffered saline without divalent ions (PBS-), sub-confluent cells were stored at -80°C for proteomic analysis and fixed in 10% formalin and embedded in paraffin for immunohistochemistry. LCN1 cells were also AMeX-fixed [16] for immunohistochemical screening. The SP2/O-Ag14 cells derived from a mouse myeloma were purchased from the RIKEN BioResource Center, and were grown in RPMI-1640 medium supplemented with 1×8-azaguanine (50× Hybri-Max, SIGMA), 10% FBS, 100 units/ml of penicillin, and 100 mg/ml of streptomycin (GIBCO).

### Cisplatin-resistant sub-lines

Cisplatin-resistant sub-lines (LCN1-cis, LC-2/ad-cis, and A549-cis) were established by culturing the cells for 6 months with Cisplatin (Randa inj., Nippon Kayaku Co., Ltd., Tokyo, Japan), starting from a concentration of 25 and rising gradually to 3,200 ng/ml. Cisplatin-resistant sub-lines were stably grown at a concentration of 3,200 ng/ml Cisplatin for over 12 months in our laboratory [17].

### Generation of monoclonal antibodies

LCN1-cis cell lysate was prepared with PBS (-) using an ultra-sonic homogenizer (UH-50; SMT Company, Tokyo, Japan). Five-week-old female BALB/c mice were immunized intra-peritoneally with 50 mg wet-weight of LCN1-cis cell lysate in 500 ml of PBS (-) 3 times with a two-week interval. The antibody titer was tested by IHC using 100-times diluted sera from the immunized mice as the first antibody on AMeX-fixed LCN1-cis cells. Three days prior to cell fusion, the animal with the highest titer was intra-peritoneally boosted by the same amount of LCN1-cis lysate. Hybridoma preparation and IHC screening with AMeX-fixed LCN1-cis cells were previously described [18,19].

### Determination of antibody isotype

An antibody, designated as KU-Lu-6, which showed membranous staining in LCN1-cis cells, but not in its parent LCN1 cells, was picked-up and further studied. To determine the isotype of the established KU-Lu-6 antibody, we used the IsoStrip^TM^ Mouse Monoclonal Antibody Isotyping Kit (Roche Diagnostics, Mannheim, Germany), according to the manufacturer’s instructions.

### Immunoprecipitation

The immunoprecipitation method used for the Pierce Protein L Agarose (Thermo Scientific, Rockford, IL, USA) in this study was according to the manufacturer’s instructions. In brief, LC-2/ad-cis cells were washed with PBS (-) and treated with radio-immunoprecipitation assay (RIPA) buffer containing Complete-mini EDTA-free (Roche Diagnostics) on ice for 30 min. After centrifugation at 15,000 rpm for 30 min at 4°C, the supernatant was collected. To conjugate the primary antibody, 200 μL of primary antibody (KU-Lu-6 hybridoma supernatant) and 50 μL of protein L agarose beads suspended in RIPA buffer were incubated with rotation at room temperature for 30 min. After centrifugation, the antibody-agarose conjugate and 500 μg of total cellular protein from the LC-2/ad-cis supernatant were incubated with rotation at room temperature for 30 min. The immunoprecipitates were collected by centrifugation at 15,000 rpm for 5 min at 4°C. After washing three times with RIPA buffer, the supernatant was carefully removed and the pellets were resuspended in 20 μL of 1×Laemmili’s buffer. Then, all of the samples were boiled and separated by SDS-PAGE with 10% polyacrylamide gel. After SDS-PAGE, gels were Zn-stained with the Negative Gel Stain MS kit (Wako Pure Chemical, Tokyo, Japan), according to the manufacturer’s instructions.

### Identification of antigen protein

The protein spot was excised from the SDS-PAGE gel and minced to 1 mm^3^, destained with destaining solution (Wako Pure Chemical), dehydrated with 100% (v/v) ACN, and dried under vacuum conditions. Tryptic digestion was performed with a minimal volume of digestion solution containing 10 ng/μl of trypsin (Trypsin Gold, Mass Spectrometry Grade, Promega Madison, WI, USA) and 25 mM NH4HCO3 for 24 hrs at 37°C. After incubation, digested protein fragments eluted in solution were collected, and gels were washed once in 5% (v/v) trifluoroacetic acid /50% (v/v) ACN and collected in the same tube.

The collected peptide fragments were analyzed using autoflex III matrix-associated laser desorption/ionization-time of flight/time of flight mass spectrometry (MALDI-TOF/TOF MS; Bruker Daltonik GmbH, Bremen, Germany). A disposable plate, spotted α-cyano-4-hydroxycinnamic acid matrix for samples, and PAC Peptide Calibstandard for calibration (Prespotted AnchorChip 96 set for Proteomics, Bruker Daltonik GmbH) were used. Peptide mass fingerprints (PMF) were measured, and then a few peaks obtained from PMF were further measured for their tandem mass spectra as parent masses. MASCOT (http://www.matrixscience.com) using the IPI Human 3.85 database (89,952 sequences; 36,291,020 residues), was used to determine proteins from PMF and tandem mass data.

Because the KU-Lu-6 antibody does not work for immunoblotting, the antibody absorption test for confirmation of the antigen using purified synthetic antigen protein was utilized.

An MYH9 expression clone was constructed from the CU013414 clone and pINSOL20 destination vector with the Gateway system (Life Technologies, USA). Protein synthesis was performed using the method in our previous report [20]. The quantification of protein was calculated as the density of the band of CBB staining in SDS-PAGE as BSA standard protein.

A hundred μL each of non-diluted hybridoma supernatants was pre-absorbed with 0.12, 0.24, and 0.48 μg of synthetic MYH9 proteins, respectively. Then, they were incubated at room temperature for 2 hrs and at 4°C overnight. After being centrifuged at 20,000 g for 10 min, each supernatant was collected and used as the first antibodies.

IHC with absorbed antibodies was described in 2.8.

### Patients and tissue specimens

A total of 266 consecutive NSCLC patients who underwent complete resection from January 2002 to December 2005 at Kitasato University Hospital were included in this retrospective cohort study. Those who received preoperative chemotherapy and/or radiotherapy were excluded. Ten percent of formalin-fixed and paraffin-embedded tissues were processed into 3-μm-thick sections and stained with hematoxylin and eosin. The histological diagnosis was based on the criteria of the World Health Organization/International Association for the Study of Lung Cancer classification of lung and pleural tumors [21]. Each patient was reassessed according to the 7th edition of the TNM classification [22]. The clinical and pathologic parameters retrospectively reviewed included the age at surgical resection, gender, smoking habits, histological type, tumor differentiation, pathological TNM (p-TNM) and stage, nodal status, intratumoral vascular invasion, intratumoral lymphatic invasion, pleural invasion, receiving adjuvant chemotherapy, viability status, and survival time after surgery. The viability status was determined based on whether or not NSCLC-related death occurred, and the survival time was defined as the duration from the date of surgery to the date of death or end of follow-up. Cases of death from other causes or lost to follow-up were treated as censored cases.

### Immunohistochemical staining of KU-Lu-6 antibody

After deparaffinizing in xylene, 3-μm thick sections or cell preparations were rehydrated in a descending ethanol series, and then treated with 3% hydrogen peroxide for 10 min. They were antigen-retrieved by autoclaving for 10 min in 0.01 M citrate buffer (pH 6.0) with 0.1% Tween20. After blocking with 2% normal swine serum/Tris-buffered saline (0.01 M Tris-HCl, pH 7.5, 150 mM NaCl) for 10 min, they were reacted with non-diluted supernatant of KU-Lu-6 antibody overnight at room temperature. After being rinsed in Tris-buffered saline three times for 5 min each, they were reacted with ChemMate ENVISION reagent (DAKO, Glostrup, Denmark) for 30 min at room temperature. They were subsequently visualized with Stable DAB solution (Invitrogen; Carlsbad, CA, USA) and counterstained with Mayer’s hematoxylin. Negative controls were prepared by substituting phosphate-buffered saline for the KU-Lu-6 antibody.

### Evaluation of immunohistochemical staining

Membranous immunostaining of tumor cells was considered to be positive for the KU-Lu-6 antibody. The staining intensity was categorized into three groups: 0 = negative; 1 = weakly positive; 2 = strongly positive. The tumor cells with a staining score of 2 were judged as positive. We observed in all tumor cells of specimen, a tumor with positive staining in more than 25% of its tumor cells was considered as positive. All of the immunostained sections were reviewed by two investigators (K.K. and S.Y.) without knowledge of the clinical data. Discordant cases were reviewed and discussed until a consensus was reached.

### Statistical analysis

Continuous variables are presented as the median (range), while numerical variables are given as N (%). The relationships between NMIIA expression and clinicopathological parameters were assessed with Pearson’s χ^2^ test or Fisher’s exact test, as appropriate. The cumulative survival of patients was estimated using the Kaplan-Meier method, and the significance of the survival differences between MYH9-positive and -negative groups was tested using the log-rank test. The 5-year cumulative survival probability was estimated using the life table method with the interval length set at 1 month. Multivariable analysis was performed by employing the Cox proportional hazards regression model to examine the interaction between MYH9 expression and other clinicopathological variables, and estimate the independent prognostic effect of MYH9 on survival by adjusting for confounding factors. The conventional *P*-value of 0.05 or less was used to determine the level of significance. All reported *P*-values are two-sided. Analyses were performed independently at our clinical research center using SPSS version 17.0 software (SPSS; Chicago, IL, USA).

## Results

### Characterization of KU-Lu-6 antibody

**Fig 1:**
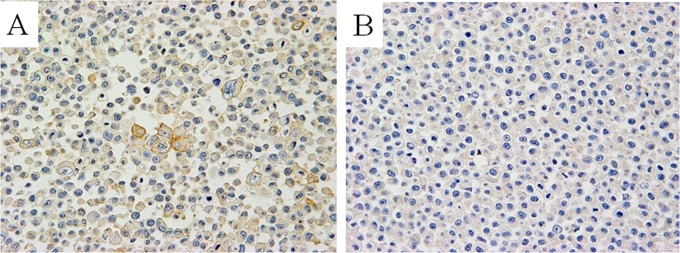
Immunohistochemical screening of the Ku-Lu-6 antibody with AMeX-fixed LCN1-cis (A) and LCN1 (B) cells. Membranous staining was observed in LCN1-cis cells, but not in LCN1 cells. (original magnification: A, B ×400).

Using AMeX-fixed LCN1-cis cells for immunohistochemical screening, we finally selected a clone designated as KU-Lu-6, which showed membranous staining on LCN1-cis cells, but not on LCN1 cells (Fig. 1).

**Fig 2:**
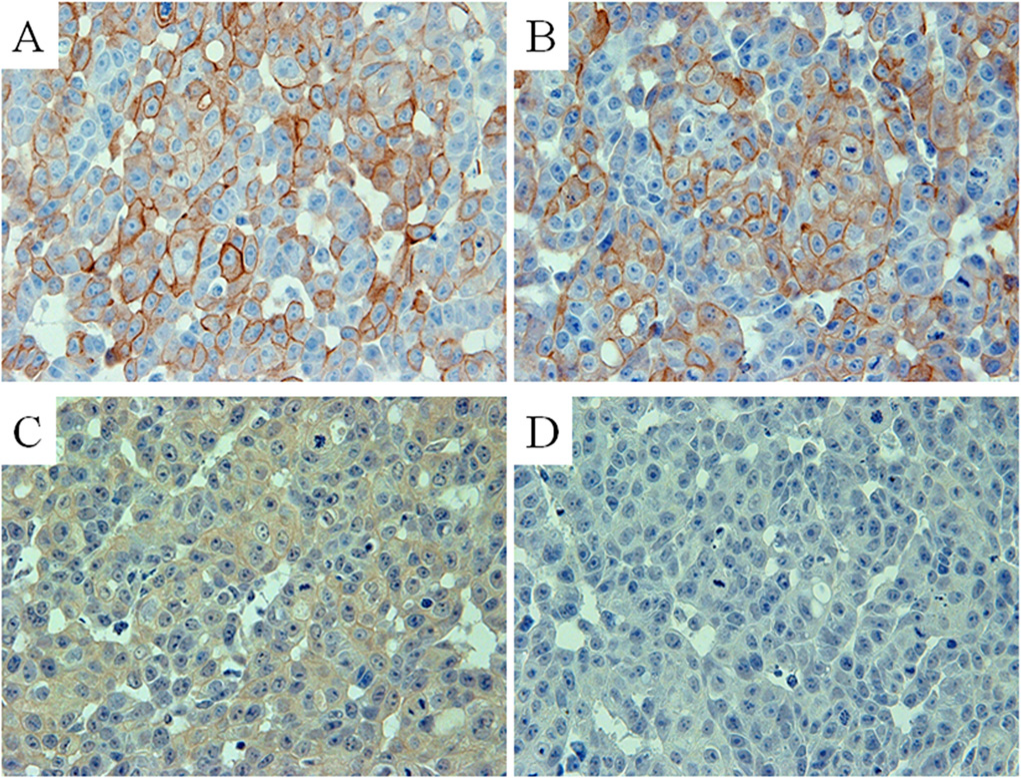
Antibody absorption test for MYH9. KU-LU-6 antibody supernatant was pre-absorbed with none (A), 0.12 ug (B), 0.24 ug (C), and 0.48 ug (D) of synthetic MYH9 proteins. Each absorbed antibody was immunostained with formalin-fixed LC2/ad-cis cells. The stainability of KU-Lu-6 antibody was gradually reduced depending on the concentration of MYH9 protein.

By immunohistochemical staining of KU-Lu-6 antibody with formalin-fixed cell lines, intensive staining was observed in cisplatin-resistant cell lines, especially in LC2/ad-cis cells (Fig. 2A).

**Fig 3:**
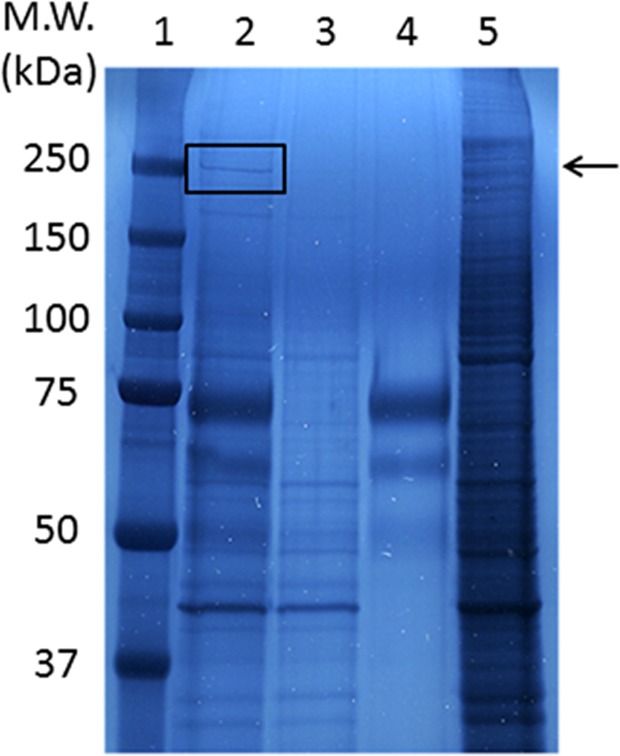
Immunoprecipitation with KU-Lu-6 antibody. Lane 1: molecular weight marker, lane 2: LC-2/ad-cis lysate combined with KU-Lu-6 antibody, lane 3: LC-2/ad-cis lysate combined with protein L, lane 4: KU-Lu-6 antibody combined with protein L, lane 5: LC-2/ad-cis lysate. Lanes 3 and 4 are negative controls, and specific immunoprecipitated product with KU-Lu-6 antibody was detected in lane 2 (arrow).

In order to identify the antigen protein recognized by the KU-Lu-6 antibody, we performed IP with lysate from LC-2/ad-cis cells. The results of IP are shown in Fig. 2. The antigenic protein was observed at roughly 250 kDa in lane 2 (Fig. 3). No signal was observed in lane 3 and 4 as a negative control (Fig. 3). To determine the antigenic protein recognized by KU-Lu-6 antibody, we excised and collected the spot from the Zn-stained gel, and proceeded with in-gel digestion. After analysis employing a MALDI-TOF/TOF MS and MASCOT search, the protein was determined as isoform 1 of myosin-9 (MYH9, accession: IPI00019502), which is composed of 1,960 amino acids with a predicted M.W. of 226,532 Da. The immunoglobulin isotype of KU-Lu-6 antibody was determined as IgM, k. By the antibody absorption test, the stainability of KU-Lu-6 antibody was gradually reduced depending on the concentration of MYH9 protein. The stainability of KU-Lu-6 antibody was completely lost with 0.48 ug of MYH9 protein (Fig. 2).

### MYH9 expression in NSCLC

**Fig 4:**
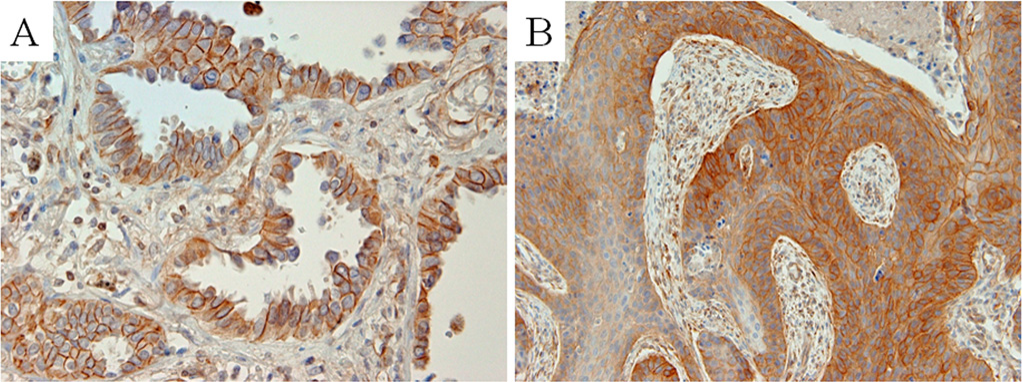
Representative immunohistochemical staining for MYH9 in NSCLC. A: MYH9 was strongly expressed in the membrane of tumor cells in adenocarcinoma. B: MYH9 was strongly expressed in the membrane and cytoplasm of tumor cells in squamous cell carcinoma. (original magnification: A, B ×400).

MYH9 was expressed in the membrane and cytoplasm of some tumor cells. Especially, the staining intensity was marked in the cell membrane. Of the 266 surgically resected NSCLCs, including 203 adenocarcinomas, 51 squamous cell carcinomas, 10 large cell carcinomas, and 2 adenosquamous cell carcinomas, positive MYH9 expression in tumor cells was observed in 102 cases (38.3%) (Fig. 4). MYH9 expression was also observed in fibroblasts in the tumor stroma and in normal bronchial epithelial cells. MYH9 expression was not detected in the normal alveolar epithelial cells. No expression was observed in the negative controls.

### Clinicopathological characteristics of patients

The clinicopathological characteristics of the patients are summarized in Table 1. A total of 168 men and 98 women were included, with ages ranging from 34 to 85 years (median, 64 years), of which 166 (62.4%) were smokers. The overall follow-up duration ranged from 3 to 129 months (median, 84 months). A total of 142 patients were alive at the end of the follow-up, 88 patients had died of lung cancer, 19 patients had died from other causes, and 17 patients were lost to follow-up. The causes of the 19 non-lung cancer deaths were pneumonia (n = 11), cholangiocellular carcinoma (n = 2), chronic obstructive pulmonary disease (n = 1), sepsis (n = 1), cerebral infarction (n = 1), acute myocardial infarction (n = 1), gastric cancer (n = 1), and leukemia (n = 1). None of these 19 patients had surgery-related deaths. In the 17 lost-to-follow-up patients, all were lost to follow-up due to discontinuing hospital attendance and were unable to be contacted. The follow-up durations of the 17 patients lost to follow-up ranged from 15 to 92 months (median, 61 months).

**Table 1:** Characteristics of the Patients.

Characteristics	Patients, N (%) (N = 266)
**Age, y**	
Median age (range)	64 (34–85)
< 65	129 (48.5)
≥ 65	137 (51.5)
**Gender**	
Male	168 (63.2)
Female	98 (36.8)
**Smoking habit**	
NS	100 (37.6)
S	166 (62.4)
**Histological type**	
AD	203 (76.3)
SQ	51 (19.2)
Others	12 (4.5)
**Tumor differentiation**	
Well/Moderately	204 (79.7)
Poorly	52 (20.3)
**p-TNM stage** *	
Stage I	153 (57.5)
Stage II	52 (19.6)
Stage III	61 (22.9)
**Receiving adjuvant chemotherapy**	
Yes	37 (13.9)
No	229 (86.1)
**Vital status**	
Alive	142 (53.4)
Lung cancer-related death	88 (33.1)
Other causes of death	19 (7.1)
Unknown	17 (6.4)

AD = adenocarcinoma; NS = never smoker; p-TNM = pathological TNM; S = smoker

SQ = squamous cell carcinoma

*Each case was reassigned to a pathological stage on the basis of the IASLC Lung Cancer Staging Project (seventh edition).

### Relationship between MYH9 expression and clinicopathological characteristics

The relationships between MYH9 expression and clinicopathological characteristics are summarized in Table 2. MYH9 expression was more frequently detected in adenocarcinoma than in squamous cell carcinoma and other histological subtypes (*P* = 0.014). MYH9 expression was also related to poorer differentiation (*P* = 0.033), intratumoral vascular invasion (*P* = 0.013), and intratumoral lymphatic invasion (*P* = 0.045). There was no significant association between MYH9 expression and the age, gender, smoking habits, p-TNM stage, nodal status, or pleural invasion.

**Table 2:** Relationships between MYH9 Expression and Clinicopathological parameters.

Clinicopathological Parameters	MYH9 Expression		Total	*P*-Value
	Positive (N = 102)	Negative (N = 164)		
**Age, y; N (%)**				.906
< 65	49 (38.0)	80 (62.0)	129	
≥ 65	53 (38.7)	84 (61.3)	137	
**Gender; N (%)**				.527
Male	62 (36.9)	106 (63.1)	168	
Female	40 (40.8)	58 (59.2)	98	
**Smoking habit; N (%)**				.865
NS	39 (39.0)	61 (61.0)	100	
S	63 (38.0)	103 (62.0)	166	
**Histological type; N (%)**				.014
AD	86 (42.6)	117 (57.4)	203	
Non-AD	16 (25.4)	47 (74.6)	63	
**Tumor differentiation; N (%)**				.033
Well/Moderately	73 (35.8)	131 (64.2)	204	
Poorly	27 (51.9)	25 (48.1)	52	
**p-TNM stage** * **; N (%)**				.050
Stage I	51 (33.3)	102 (66.7)	153	
Stage II/III	51 (45.1)	62 (54.9)	113	
**Nodal status; N (%)**				.093
N0	64 (35.0)	119 (65.0)	183	
N1/N2/N3	38 (45.8)	45 (54.2)	83	
**Vascular invasion; N (%)**				.013
Yes	55 (48.2)	59 (51.8)	114	
No	34 (29.6)	81 (70.4)	115	
**Lymphatic invasion; N (%)**				.045
Yes	40 (44.0)	51 (56.0)	91	
No	35 (30.4)	80 (69.6)	115	
**Pleural invasion; N (%)**				.490
Yes	41 (41.0)	59 (59.0)	100	
No	61 (36.7)	105 (63.3)	166	

* See Table 1 for explanation of abbreviations and note on asterisk.

### Kaplan-Meier estimate of survival in MYH9-positive and -negative patients

**Fig 5:**
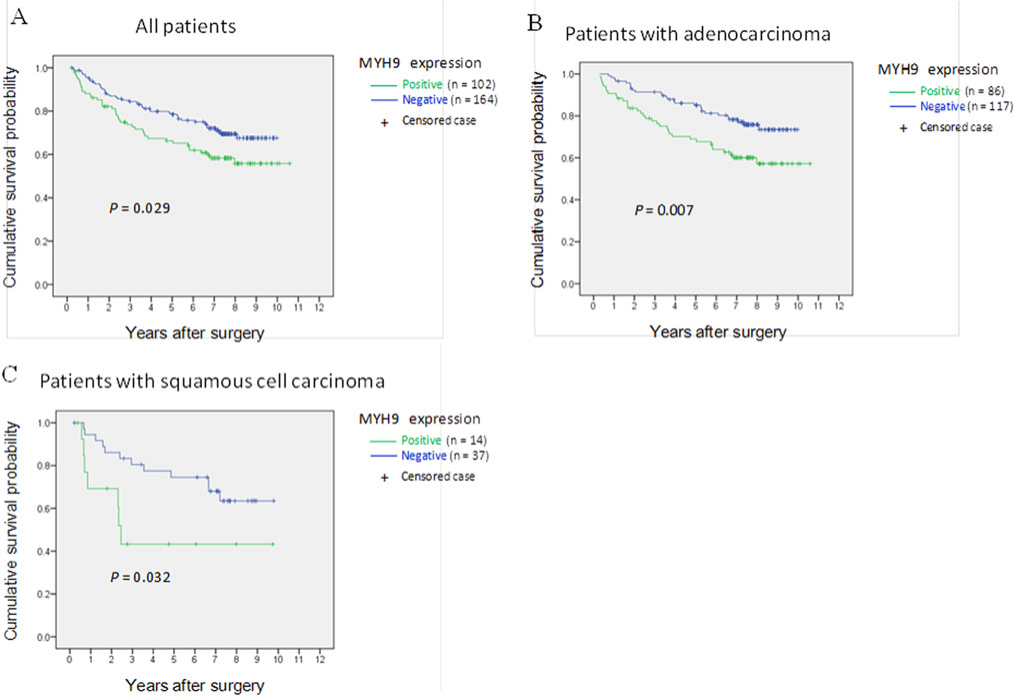
Cumulative survival of patients with NSCLC according to MYH9 expression estimated by the Kaplan-Meier method. (A) for all patients; (B) for patients with adenocarcinoma; (C) for patients with squamous cell carcinoma, treating all other causes of death and lost to follow-up as censored cases. MYH9 expression was significantly correlated with poorer survival in resected NSCLCs.

All patients were included in survival analysis. The overall follow-up periods ranged from 3 to 129 months (median, 84 months), and the 5-year cumulative survival probability was 70% for all patients. Because a cumulative survival probability of 50% had not yet been reached, the overall median survival time was not determined. The 5-year cumulative survival probability was 66% for the MYH9-positive group and 78% for the MYH9-negative group. While the median survival time was not available, the survival rate of MYH9-positive group was significantly poorer group (*P* = 0.029) (Fig. 5A). In further analyses, MYH9 expression was significantly correlated with poorer survival in patients with either adenocarcinoma (*P* = 0.007) (Fig. 5B) or squamous cell carcinoma (*P* = 0.032) (Fig. 5C). The 5-year survival probability was 69 and 85% for MYH9-positive and -negative adenocarcinoma patients, respectively, and 43 and 74% for MYH9-positive and -negative squamous cell carcinoma patients, respectively.

### Effect of MYH9 expression on survival with uni- and multivariable analyses

Univariable analysis was performed according to the Cox proportional hazard model to evaluate the effect of MYH9 expression and other clinicopathological factors on survival. The results indicated that the histological type (HR, 1.94; 95% CI, 1.23–3.06; *P* = 0.004), p-TNM stage (HR, 4.58; 95% CI, 2.89–7.26; *P* < 0.001), adjuvant chemotherapy (HR, 2.81; 95% CI, 1.73–4.57; *P* < 0.001), tumor differentiation (HR, 2.45; 95% CI, 1.53–3.92; *P* < 0.001), vascular invasion (HR, 5.67; 95% CI, 3.25–9.90; *P* < 0.001), lymphatic invasion (HR, 4.43; 95% CI, 2.65–7.40; *P* < 0.001), pleural invasion (HR, 2.64; 95% CI, 1.73–4.03; *P* < 0.001), and MYH9 expression (HR, 1.57; 95% CI, 1.03–2.39; *P* = 0.03) were significant predictors of cancer-specific survival. Furthermore, MYH9 expression and other clinicopathlogical variables including the histological type, p-TNM stage, adjuvant chemotherapy, tumor differentiation, vascular invasion, lymphatic invasion, and pleural invasion were entered into multivariable analysis using the Cox proportional hazards regression model. The results indicated that MYH9 expression was a significantly independent predictor of a poorer survival (HR, 2.15; 95%CI, 1.17–3.92; *P* = 0.01) (Table 3).

**Table 3:** Uni- and Multivariable Analyses of the Effect of MYH9 Expression on Survival.

Factors	Univariable Analysis			Multivariable Analysis		
	HR	95% CI	*P*-Value	HR	95% CI	*P*-Value
**MYH9 expression**						
Positive vs. Negative	1.57	1.03–2.39	0.03	2.15	1.17–3.92	0.01
**Age**						
≥ 65 vs. < 65	1.11	0.73–1.69	0.60	−	−	−
**Gender**						
Male vs. Female	1.39	0.89–2.18	0.14	−	−	−
**Smoking habit**						
Smokers vs.Never Smokers	1.41	0.91–2.20	0.12	−	−	−
**Histological type**						
Non-AD vs. AD	1.94	1.23–3.06	0.004	0.50	0.23–1.09	0.08
**p-TNM stage**						
Stage II/III vs. Stage I	4.58	2.89–7.26	< 0.001	2.23	1.13–4.40	0.02
**Adjuvant chemotherapy**						
No vs. Yes	2.81	1.73–4.57	< 0.001	3.55	1.78–7.07	<0.001
**Tumor differentiation**						
Poorly vs. Well/Moderately	2.45	1.53–3.92	< 0.001	2.21	1.14–4.26	0.01
**Vascular invasion**						
Yes vs. No	5.67	3.25–9.90	< 0.001	1.62	0.70–3.72	0.25
**Lymphatic invasion**						
Yes vs. No	4.43	2.65–7.40	< 0.001	1.46	0.72–2.94	0.28
**Pleural invasion**						
Yes vs. No	2.64	1.73–4.03	< 0.001	1.99	1.09–3.64	0.02

Analyses were performed using Cox proportional hazard regression.

AD = adenocarcinoma; HR = hazard ratio.

## Discussion

In the present study, MYH9 was expressed in 102 of 266 (38.3%) NSCLCs, and its expression was significantly correlated with an adenocarcinoma histology, poorer differentiation, and intratumoral vascular and lymphatic invasion. In agreement with previous reports of esophageal [11], bladder [12] and gastric cancer[13], the present study revealed that MYH9 expression is an independent prognostic factor and associated with a poorer prognosis of patients with resected NSCLCs. Although Maeda et al. reported that patients with stage I lung adenocarcinoma lacking the expression of either MYH9 or vimentin show a favorable outcome without postoperative adjuvant chemotherapy [14], the present study revealed that MYH9 expression is an independent prognostic factor for survival in patients with resected NSCLCs including pathological stage I-III and lung squamous cell carcinoma.

In the present study, MYH9 staining was detected in both the membrane and cytoplasm of some tumor cells, although the staining intensity was markedly stronger in the cell membrane. In MDA-MB-231 breast cancer cells spreading on fibronectin, MYH9 is observed at the marginal spreading lamellar region of the cells [9]. Because MYH9 is reported to contribute to cell polarity and lamellipodia at the cell periphery and leading edge, strong staining intensity at the cell membrane may reflect the MYH9 activity in cancer cell migration and invasion.

Local microenvironment invasion, such as intratumoral lymphovascular permeation, is an important step in the early stage of tumor metastasis. MYH9 contributes to cell migration which is required for invasion of the local microenvironment. *In vitro*, several studies reported that MYH9-depleted cells showed defective migration [10,11]. Furthermore, lamellipodia formation at the leading edge of the cell is necessary for migration [23], and such formation is controlled by Rac, the WAVE complex, and Arp2/3complex [24]. Recently, Morimura et al. reported that MYH9 is also necessary for lamellipodia formation by binding to WAVE2 [25]. Thus, MYH9 expression may be associated with the acquisition of migration and invasion capabilities of tumor cells, which subsequently results in highly intratumoral lymphovascular invasion, and poorer prognoses in the present study.

In the present study, MYH9 expression was significantly correlated with poorer tumor differentiation. Poorly differentiated carcinoma is characterized by the facilitation of cytokinesis. In cytokinesis, cells build a contractile ring that constricts the plasma membrane to generate two daughter cells connected by a cytoplasmic bridge. MYH9 contributes to contraction of the contractile ring and plays a key role in cytokinesis [26,27]. Moreover, a previous study reported that treatment with blebbistatin, an inhibitor of MYH9, leads to the failure of cytokinesis [28]. Thus, it is not surprising that MYH9 expression is associated with poorer tumor differentiation in the present study.

## Conclusion

We have reported that MYH9 is expressed in a subset of NSCLC, and its expression is related to the adenocarcinoma histology, poorer differentiation, intratumoral vascular invasion, intratumoral lymphatic invasion, and a poorer prognosis. MYH9 expression is an independent prognostic factor for survival in patients with resected NSCLCs, although its prognostic significance still requires confirmation with larger patient populations.
